# Categorical and Geographical Separation in Science

**DOI:** 10.1038/s41598-018-26511-4

**Published:** 2018-05-29

**Authors:** Julian Sienkiewicz, Krzysztof Soja, Janusz A. Hołyst, Peter M. A. Sloot

**Affiliations:** 1 Faculty of Physics, Center of Excellence for Complex Systems Research, Warsaw University of Technology, Koszykowa 75, Warsaw, 00662 Poland; 2 National Research University ITMO, 49 Kronverkskiy av., Saint Petersburg, 197101 Russia; 3 Institute for Advanced Study, University of Amsterdam, Oude Turfmarkt 147, Amsterdam, 1012 GC The Netherlands; 4 Complexity Institute, Nanyang Technological University, 61 Nanyang Drive, Singapore, 637335 Singapore

## Abstract

We study scientific collaboration at the level of universities. The scope of this study is to answer two fundamental questions: (i) can one indicate a category (i.e., a scientific discipline) that has the greatest impact on the rank of the university and (ii) do the best universities collaborate with the best ones only? Restricting ourselves to the 100 best universities from year 2009 we show how the number of publications in certain categories correlates with the university rank. Strikingly, the expected negative trend is not observed in all cases – for some categories even positive values are obtained. After applying Principal Component Analysis we observe clear categorical separation of scientific disciplines, dividing the papers into almost separate clusters connected to natural sciences, medicine and arts and humanities. Moreover, using complex networks analysis, we give hints that the scientific collaboration is still embedded in the physical space and the number of common papers decays with the geographical distance between them.

## Introduction

The idea of so-called *science of science* is not entirely new: 20th century is well known for its critical works of Kuhn^1^, Popper^2^, Lakatos^3^ and Feyerabend^4^ who tried to build models describing how science should work or, which is far more important, to show how it in fact *does* work. However it is only in recent times that, owing to the start of the era of overwhelming data, it is now possible to track this problem quantitatively^5,6^. Several studies are on a journey to answer such intriguing questions like “Who is the best scientist?”, “What makes the best university” etc^7–14^.

There are at least three separate factors that can be regarded as key components of today’s science and the way it is recognized: papers, citations and rankings. The last one is devoted rather to whole unities like universities or departments although recent studies consider it also in the scope of individuals^14^. It has been argued that rankings still can be perceived as not enough deep measures “providing finalized, seemingly unrelated indicator values”^15^. On the other hand it is well known that scientific impact is a multi-dimensional construct and that using a *single* measure is not advisable^16^.

Nonetheless, rankings are clearly a derivative of the number of published papers. However apart from just raw numbers the quality of science comes often with two additional factors: specialization and collaboration. Interestingly the type of the scientific category can dramatically change both the way the paper is written and received, e.g., in the case of simple lexical factors as title length its impact on the acquired citations change significantly from one category to another^17^. In the same manner it is possible to spot that the number of citations per paper can vary by several orders of magnitude and are highest in multidisciplinary sciences, general internal medicine, and biochemistry and lowest in literature, poetry, and dance^18^. These studies can go even as deep as to fascinating notion of *scientific meme* propagating along the citation graph^19,20^.

Collaboration has been in the scope of interest for a long time^21,22^ and it is generally considered that it leads to high impact publications^23^. One of recognized factors affecting the level of collaboration is undoubtedly geographic proximity: usually one expects to find a decaying probability of citation as well as common papers with distance^24,25^, however it can also be connected to such features as ethnicity or level of economic development^26^.

In this study we perform an investigation for a selected group of 100 best universities to unravel how the scientific productivity measured in the number of published papers per scientific categories (e.g, physics, art etc) correlates with the rank of the university. Using Principal Component Analysis (PCA) we study whether scientific categories coming from different areas (natural science, humanities etc) tend to stick together. In the second part of the paper we examine the complex network^27^ of scientific collaboration among 100 best universities and study the properties of such a network using the concept of weight threshold^28^.

## Results

We use the QS World University Ranking and service Web of Science datasets to examine patterns of category and geographic separation (see Methods for details). The data describes 100 best universities in a form of two matrices **P**_*ij*_ (100 universities by 181 categories) and **C**_*ij*_ (100 by 100 universities). The first matrix contains information about the number of papers published by a specific university *i* in a given scientific category *j* while the second one stores the total number of common papers among universities *i* and *j* (regardless of the category).

The main text of this paper concerns absolute numbers of quantities **P**_*ij*_ and **C**_*ij*_ while the Supplementary Information contains some results for the scaled cases.

### Rank–number correlations for categories

**Figure 1:**
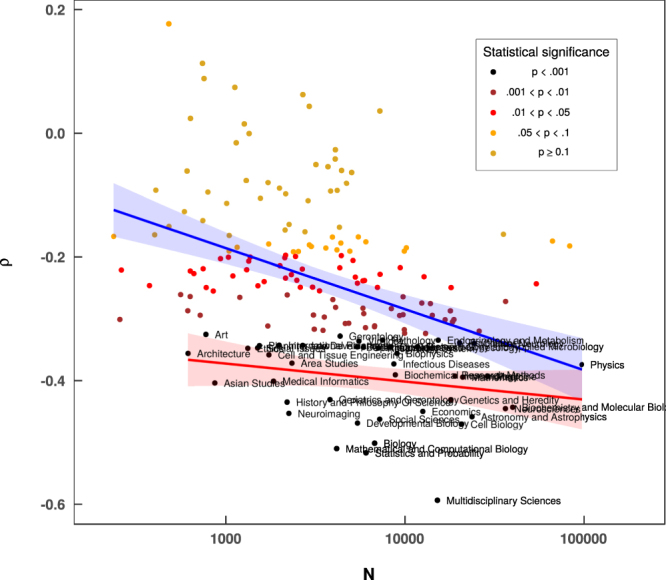
Correlations coefficients. Each data point represents a separate scientific category and gives the Spearman’s correlation coefficient *ρ* between the rank of the university and the ranked number of papers *N* in this category (shown as X-axis). The colors reflect statistical significance of the measure (see legend) and category names are shown only for the most significant points (p-value < 0.001). Solid lines represent log-linear fits to all points (blue) and most significant points (p-value < 0.001, red). Shades surrounding the lines represent 95% confidence interval.

It is interesting to understand how the university rank correlates with the number of scientific publications and, which is even far more intriguing, to split these relations according to different scientific categories. Naively one would expect a strong *negative* correlation between these quantities as larger number of papers should be reflected in acquiring higher rank (thus smaller number). The results for our data analysis are shown in Tables 1, 2 and Fig. 1, where we plot correlation coefficient *ρ* against the total number of papers *N* published in the given category (an alternative and much more straightforward method would be to use regression analysis however, in this case, it brings unreliable results - see SI for details). In each case *ρ* was obtained by taking one of the columns *j* of matrix *P*_*ij*_, ranking it and correlating with the university rank, thus calculating Spearman’s rank correlation coefficient. The outcome clearly suggests that there are categories for which we observe even *positive* correlation coefficient. On the other hand, one has to take into account the fact that in these cases statistical significance of such results is usually very low (p-value > 0.05) as depicted in Fig. 1. When treated as a whole the data points give evidence of a log-linear relationship *ρ* = *a* + *b* log *N* (blue solid line in Fig. 1) between correlation coefficient and the number of papers with *a* = 0.098 ± 0.056 (*p* = 0.08) and *b* = −0.0415 ± 0.0068 (*p* < 0.001). A similar fit performed only for the highly significant categories (red solid line in Fig. 1) yields *a* = −0.285 ± 0.072 (*p* < 0.001) and *b* = −0.0127 ± 0.0081 (*p* = 0.13). An insignificant value of *b* in this case means that the level of correlations for the selected group of categories is in fact constant, contrary to the previous situation where we observe a significant decrease with *N*. It is worth to mention here that using not absolute but relative numbers of papers (i.e., divide by the total number of papers from a given university) leads to different results where positive correlations for certain categories are significant (see Fig. S1 in Supplementary Information). Interestingly, the category of *Multidisciplinary Sciences* seems to be unexpectedly robust, regardless of the method used (cf Fig. 1 and S1 in SI) it yields the highest correlation value, which might suggest that interdisciplinary research has a substantial influence on university ranking.

**Table 1:** Correlation coefficients in categories.

Category	*N*	*ρ*	Category	*N*	*ρ*
Acoustics	2997	−0.183	Agricultural Economics and Policy	262	−0.221*
Agricultural Engineering	480	0.177	Agriculture	2921	0.044
Agronomy	1267	0.015	Allergy	2539	−0.191
Anatomy and Morphology	1096	−0.231*	Andrology	257	−0.301**
Anesthesiology	2602	−0.249*	Anthropology	3535	−0.297**
Archaeology	1341	−0.207*	Architecture	616	−0.356***
Area Studies	2337	−0.371***	Art	775	−0.325***
Asian Studies	869	−0.403***	Astronomy and Astrophysics	23507	−0.458***
Automation and Control Systems	5809	−0.238*	Behavioral Sciences	5393	−0.345***
Biochemical Research Methods	8789	−0.390***	Biochemistry and Molecular Biology	39647	−0.442***
Biodiversity Conservation	1509	−0.247*	Biology	6769	−0.501***
Biophysics	8981	−0.356***	Biotechnology and Applied Microbiology	11698	−0.344***
Business	6739	−0.313**	Cardiac and Cardiovascular Systems	17817	−0.287**
Cell Biology	20596	−0.470***	Cell and Tissue Engineering	1738	−0.358***
Chemistry	65996	−0.174.	Classics	745	−0.141
Clinical Neurology	24176	−0.339***	Communication	1558	−0.105
Computer Science	53600	−0.243*	Construction and Building Technology	2157	−0.098
Criminology and Penology	748	−0.219*	Critical Care Medicine	3945	−0.269**
Crystallography	2690	0.062	Dance	17	−0.072
Demography	614	−0.287**	Dentistry	4079	−0.042
Dermatology	5267	−0.232*	Developmental Biology	5417	−0.468***
Ecology	9358	−0.217*	Economics	12516	−0.449***
Education	2488	−0.238*	Education and Educational Research	4373	−0.178
Electrochemistry	2876	−0.109	Emergency Medicine	2003	−0.214*
Endocrinology and Metabolism	15241	−0.334***	Energy and Fuels	4709	−0.081
Engineering	82305	−0.182	Entomology	1348	−0.000
Environmental Sciences	12350	−0.274**	Environmental Studies	3078	−0.294**
Ergonomics	634	0.024	Ethics	1325	−0.347***
Ethnic Studies	483	−0.151	Evolutionary Biology	5809	−0.283**
Family Studies	1198	−0.265**	Film	376	−0.246*
Fisheries	1122	0.074	Folklore	91	−0.114
Food Science and Technology	4087	−0.027	Forestry	1299	−0.076
Gastroenterology and Hepatology	9901	−0.323**	Genetics and Heredity	17932	−0.430***
Geochemistry and Geophysics	9285	−0.295**	Geography	4426	−0.060
Geology	1719	−0.080	Geosciences	10126	−0.185
Geriatrics and Gerontology	3801	−0.430***	Gerontology	4331	−0.328***
Health Care Sciences and Services	6751	−0.311**	Health Policy and Services	4840	−0.307**
Hematology	18635	−0.301**	History	7000	−0.249*
History Of Social Sciences	852	−0.255*	History and Philosophy Of Science	2196	−0.434***
Horticulture	755	0.088	Hospitality	740	0.113
Humanities	3110	−0.317**	Imaging Science and Photographic Technology	2152	−0.234*
Immunology	18895	−0.392***	Industrial Relations and Labor	664	−0.227*
Infectious Diseases	8625	−0.373***	Information Science and Library Science	2132	−0.201*
Instruments and Instrumentation	5474	−0.168	Integrative and Complementary Medicine	634	−0.223*
International Relations	1983	−0.342***	Language and Linguistics	2253	−0.148
Law	2684	−0.343***	Limnology	1012	−0.113
Linguistics	2670	−0.220*	Literary Reviews	633	−0.264**

**Table 2:** Correlation coefficients in categories (ctnd).

Category	*N*	*ρ*	Category	*N*	*ρ*
Literary Theory and Criticism	560	−0.261**	Literature	4158	−0.189
Management	5410	−0.242*	Marine and Freshwater Biology	3182	−0.051
Materials Science	35196	−0.163	Mathematical and Computational Biology	4155	−0.510***
Mathematics	20834	−0.394***	Mechanics	7236	−0.228*
Medical Ethics	778	−0.249*	Medical Informatics	1845	−0.401***
Medical Laboratory Technology	1635	−0.240*	Medicine	28662	−0.393***
Medieval and Renaissance Studies	720	−0.294**	Metallurgy and Metallurgical Engineering	4295	−0.152
Meteorology and Atmospheric Sciences	6003	−0.314**	Microbiology	9708	−0.264**
Microscopy	607	−0.061	Mineralogy	1307	−0.221*
Mining and Mineral Processing	795	−0.095	Multidisciplinary Sciences	15175	−0.594***
Music	935	−0.203*	Mycology	588	−0.127
Nanoscience and Nanotechnology	12710	−0.232*	Neuroimaging	2247	−0.452***
Neurosciences	36120	−0.445***	Nuclear Science and Technology	3605	−0.186
Nursing	2923	−0.181	Nutrition and Dietetics	5111	−0.206*
Obstetrics and Gynecology	8228	−0.345***	Oceanography	2763	−0.159
Oncology	25768	−0.320**	Operations Research and Management Science	4088	−0.281**
Ophthalmology	5846	−0.346***	Optics	13796	−0.275**
Ornithology	408	−0.092	Orthopedics	4399	−0.198*
Otorhinolaryngology	2320	−0.228*	Paleontology	1733	−0.179.
Parasitology	2200	−0.261**	Pathology	7470	−0.334***
Pediatrics	9863	−0.317**	Peripheral Vascular Disease	14139	−0.294**
Pharmacology and Pharmacy	17978	−0.249*	Philosophy	2381	−0.192.
Physics	96469	−0.374***	Physiology	9709	−0.293**
Planning and Development	1539	−0.343***	Plant Sciences	7240	0.036
Poetry	237	−0.167	Political Science	4627	−0.307**
Polymer Science	4909	−0.191	Psychiatry	20036	−0.338***
Psychology	36186	−0.272**	Public	18308	−0.305**
Public Administration	1046	−0.165	Radiology	12963	−0.323**
Rehabilitation	3833	−0.094	Religion	2140	−0.157
Remote Sensing	1367	−0.200*	Reproductive Biology	4315	−0.217*
Respiratory System	7071	−0.347***	Rheumatology	5928	−0.238*
Robotics	2447	−0.199*	Social Issues	1503	−0.347***
Social Sciences	7206	−0.462***	Social Work	1035	−0.201*
Sociology	3477	−0.319**	Soil Science	1142	−0.015
Spectroscopy	3043	−0.249*	Sport Sciences	4172	−0.093
Statistics and Probability	6058	−0.517***	Substance Abuse	3324	−0.255*
Surgery	16669	−0.301**	Telecommunications	9861	−0.191.
Theater	402	−0.164	Thermodynamics	2154	−0.197*
Toxicology	3923	−0.168	Transplantation	5870	−0.292**
Transportation	1158	−0.184	Transportation Science and Technology	1991	−0.089
Tropical Medicine	1714	−0.312**	Urban Studies	1044	−0.191
Urology and Nephrology	8348	−0.283**	Veterinary Sciences	5039	−0.063
Virology	5516	−0.336***	Water Resources	3716	−0.054
Zoology	6031	−0.176

### Categorical separation

**Figure 2:**
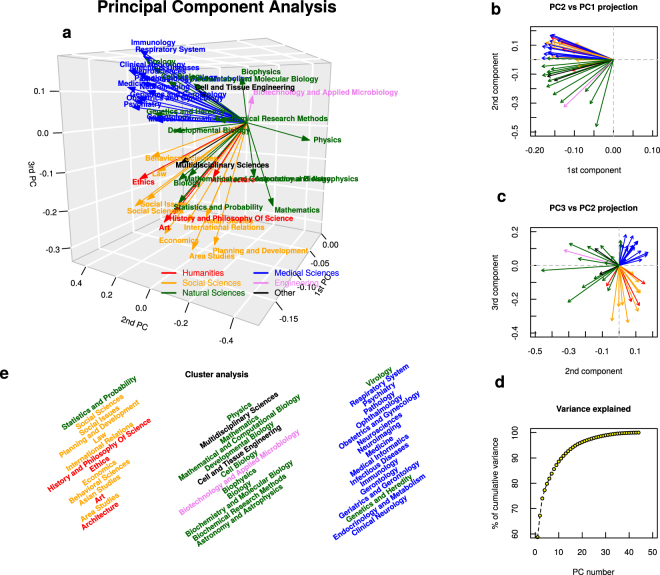
Principal Component Analysis (PCA) of scientific category data. Given the number of papers each of the 100 universities published in 44 different scientific categories (chosen according to results obtained in Fig. 1) we perform Principal Component Analysis. Panel (a) presents the outcome for three most important principal components: each arrow represents the position of an original category (e.g., *Physics*, *Multidisciplinary Sciences*) in the new set coordinates. The colors of arrows are connected to the OECD classification^29^ (see legend). Panels (b) and (c) show the projection of PCA results onto, respectively, 2nd PC — 1st PC and 3rd PC **–** 2nd PC planes. Panel (d) presents the cumulative value of variance explained by the consecutive PCs. Panel (e) shows the outcomes of cluster analysis (k-means algorithm) for the results obtained by PCA (we set the number of clusters to 3).

As a next step of our analysis, we check the hypothesis of categorical separation of science. In order to test this assumption we perform a Principal Component Analysis (PCA) for matrix **P**_*ij*_ where we restrict ourselves to those categories that were identified as highly correlated ones (see Fig. 1). Figure 2 presents the results of this PCA: the main panel (Fig. 2a) shows a 3D projection of the original 44 categories onto the first three principal components. As can be seen in Fig. 2d, the first three principal components explain around 75% of data variability. Each category was marked with a color connected to its OECD classification^29^ that contains six different areas: *Natural Sciences*, *Engineering and Technology*, *Medical & Health Sciences*, *Agricultural Sciences*, *Social Sciences* and *Humanities*, marking with a different color the scientific category *Multidisciplinary Sciences*. The 3D plot suggests two separate bundles of categories — one connected to medical sciences combined with complementary natural sciences (such as *Virology* or *Cell Biology*) and the second identified as mainly social sciences and humanities. Interestingly, such core natural sciences like *Physics* and *Mathematics* tend to point in directions separated from these two bundles. The other intriguing fact is almost complete absence of agricultural and engineering sciences (except for one category) in this scheme. Another typical way often used to present the results of PCA is to show them in a form of so-called bi-plot, i.e., two dimensional projections of consecutive PCs. Figure 2b,c provides this additional information: the values of the first PC are if the same sign, while the 2nd PC differentiates between natural sciences and other. It is Fig. 2c that uncovers a very clear distinction among natural sciences, medical sciences and social sciences with humanities. This distinction comes also in a clear way from the cluster analysis — Fig. 2e provides results from k-means algorithm used in case of the outcomes from PCA. When searching for three clusters we obtain almost perfect separation among natural sciences, medicine and humanities and social sciences.

### Network analysis

**Figure 3:**
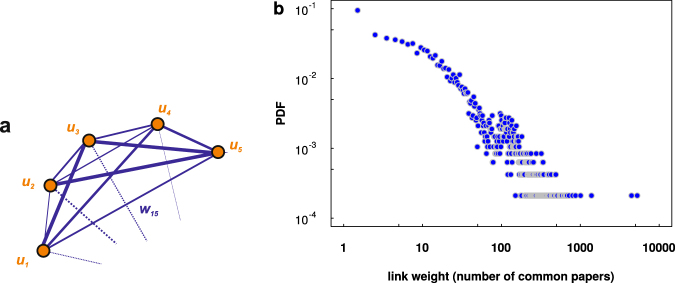
(**a**) Representation of the university collaboration network. Each node is a university and links show the connections between them. The width of each link corresponds to the number of common publications between the nodes in question. (**b**) Link weight probability distribution function (PDF).

Apart from the categorical point of view we can also consider university quality by analyzing the direct connections between universities *i* and *j* on the basis of the collaboration matrix **C**_*ij*_ where the element **C**_*ij*_ gives the number of common publications of institutions *i* and *j*. The structure of such a collaboration network is depicted in Fig. 3a where each node (vertex) is a university and links (edges) show the connections between them. The width of each link corresponds to the number of common publications between the universities. The algorithm used to obtain this structure is the following. Using 100 highest ranked universities, for each of them (*u*_1_, *u*_2_, …, *u*_100_) we search for its publications *p*_1_, *p*_2_, …, *p*_*M*(*u*1)_. Then, if among the co-authors of *p*_1_ there is any that comes from either of the universities *u*_2_, …, *u*_100_ a link of weight *w* = 1 between those universities (e.g., *u*_1_ and *u*_2_) is established. The weight is increased by one each time *u*_2_ is found among the following publications of *u*_1_. Finally the weight of the link between nodes *u*_1_ and *u*_2_ is just the number of their common publications (as seen in the database).

#### Weights probability distribution

In order to examine the fundamental properties of the weighted network of collaboration we need to compute link weight probability distribution function (PDF) which can give an idea about the diversity of number of publications between universities. Figure 3b presents link weight PDF, suggesting a fat-tail distribution where the majority of link weights can be found between *w* = 1 and *w* = 10.

#### Weight threshold

**Figure 4:**
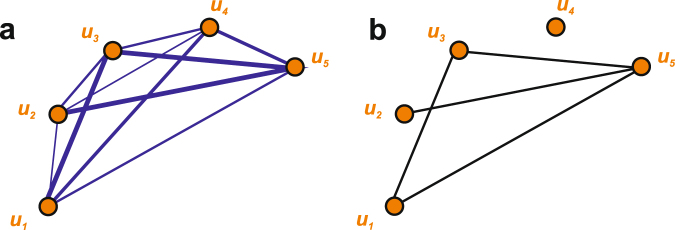
Illustration of the weight threshold concept: (**a**) a weighted university network with weights proportional to the number of common publications, (**b**) an unweighted network constructed from the weighted network of panel (a) by imposing a weight threshold — only links with weights *w* > *w*_*T*_ are kept.

In the following analysis will use the concept of weight threshold^28^ depicted in Fig. 4. Let us take the original network of 5 fully connected universities seen in Fig. 4a and assume now that we are interested in constructing an unweighted network that would take into account only the connections with weight higher than a certain threshold weight *w*_*T*_ (*w* > *w*_*T*_). A possible outcome of this procedure is presented in Fig. 4b - all the links with *w* < *w*_*T*_ are omitted and as a result we obtain a network where links indicate only connections between nodes (i.e., they do not have any value).

Using weight threshold as a parameter it is possible to obtain several unweighted networks - for each value of *w*_*T*_ in the range 〈*w*_*min*_; *w*_*max*_〉 we get a different network *NT*(*w*_*T*_) whose structure is determined only by *w*_*T*_. Then, for each of these networks it is possible to compute standard network quantities: (i) number of nodes *N* that have a at least one link (i.e., nodes with degree *k*_*i*_ = 0 are not taken into account), (ii) Number of edges (links) *E* between the nodes, (iii) the average shortest path 〈*l*〉, (iv) clustering coefficient *C*, (v) assortativity coefficient *r* (vi) size *S* of largest connected component with number *n* of components (see Materials and Methods for details).

#### Network observables as a function of weight threshold

**Figure 5:**
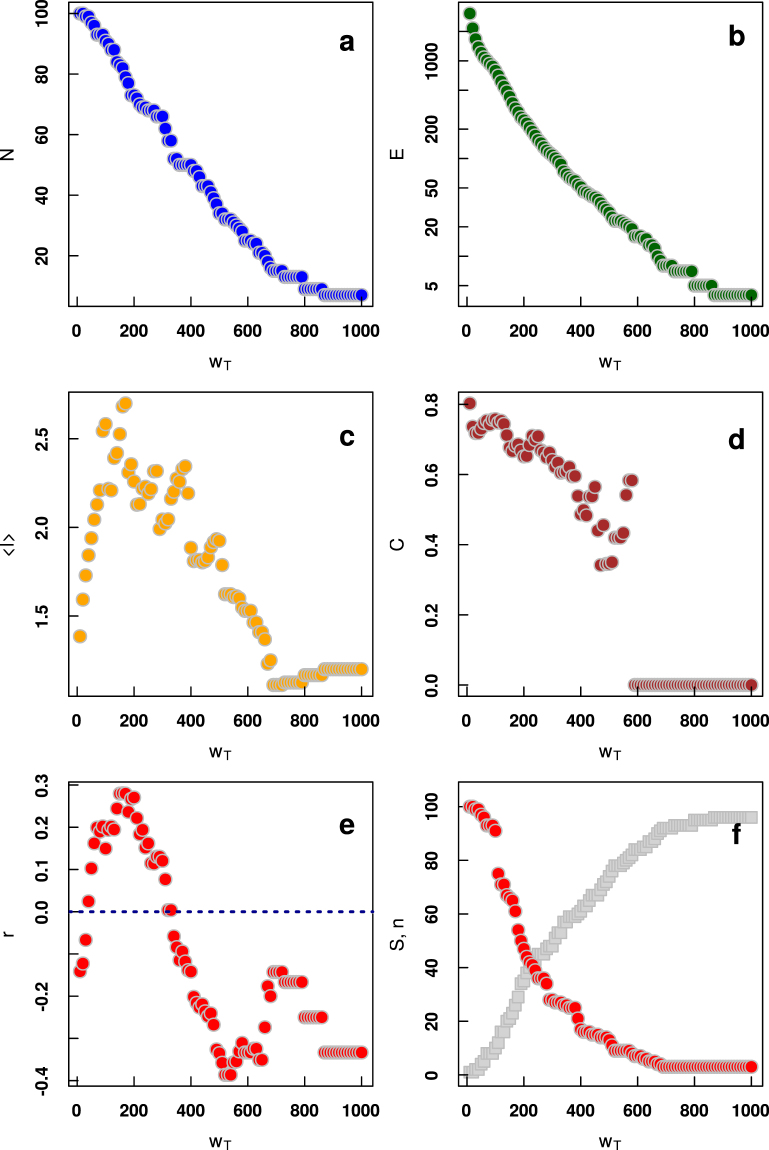
Comparison of collaboration networks observable as functions of weight threshold *w*_*T*_: (**a**) number of nodes *N* (**b**) number of edges *E*, (**c**) average shortest path 〈*l*〉, (**d**) clustering coefficient *C*, (**e**) assortativity coefficient *r*, (**f**) size of the largest connected component *S* (red points) and number of components *n* (grey points).

Figure 5 depicts the above described network parameters as a function of the weight threshold *w*_*T*_. First, as can be seen in Fig. 5a, the number of nodes *N* is a linearly decreasing function of the weight threshold *w*_*T*_. The number edges *E* decreases faster, following an exponential function (Fig. 5b). On the other hand the average shortest path 〈*l*〉 (Fig. 5c) is a non-monotonic function of weight threshold, reaching its peak for *w*_*T*_ ≈ 200. Clustering coefficient *C* (Fig. 5d) decreases with weight threshold up to the point *w*_*T*_ ≈ 500 where it rapidly drops down to 0. The most interesting is the behavior of *r*(*w*_*T*_) shown in Fig. 5e: the coefficient starts with *r* < 0, while for larger thresholds it crosses *r* = 0 and for *w*_*T*_ ≈ 200 it takes its maximal value. Then once again it drops down below zero reaching *r* ≈ −0.4 for *w*_*T*_ around 500. Finally it increases toward zero for larger *w*_*T*_. In the case of largest connected component *S* Fig. 5f) we observe a series of rapid decreases, e.g., for *w*_*t*_ ≈ 100 where *S* drops down by 20%. These results are quantitatively different from the ones obtained by randomly reshuffling the weights of the network (see SI for details).

#### Network visualisation

**Figure 6:**
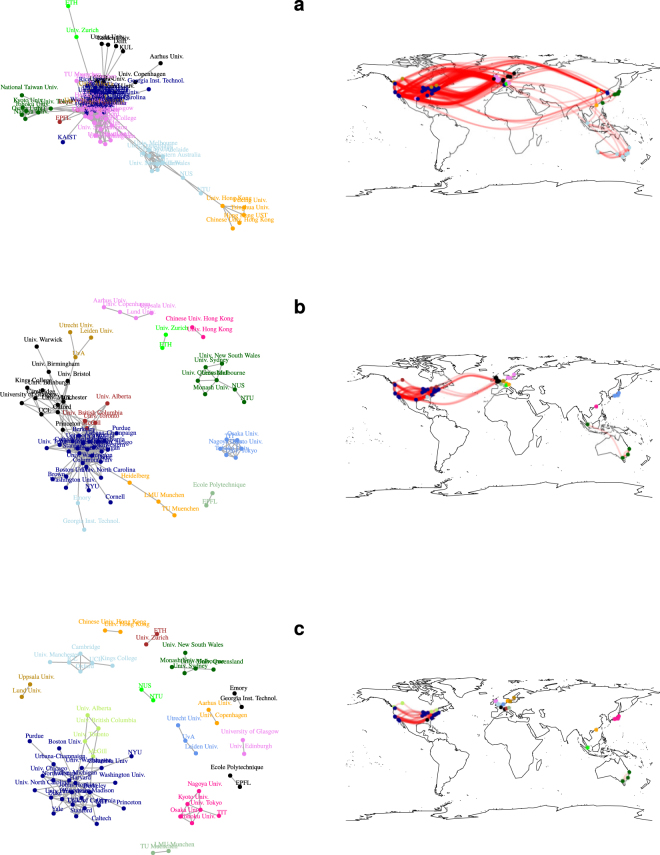
Snapshots of network topology for different thresholds: (**a**) *w*_*T*_ = 100, (**b**) *w*_*T*_ = 200 and (**c**) *w*_*T*_ = 300. The colors of vertices correspond to the assignment from a community detection algorithm (fast greedy modularity optimization algorithm^47^) and therefore they can change from one panel to another. Plots were created combining open-source packages ${\mathtt{i}}{\mathtt{g}}{\mathtt{r}}{\mathtt{a}}{\mathtt{p}}{\mathtt{h}}$^31^ (nodes and links) and ${\mathtt{m}}{\mathtt{a}}{\mathtt{p}}{\mathtt{s}}$^48^ (world map) for ${\mathtt{R}}$ language^30^.

**Figure 7:**
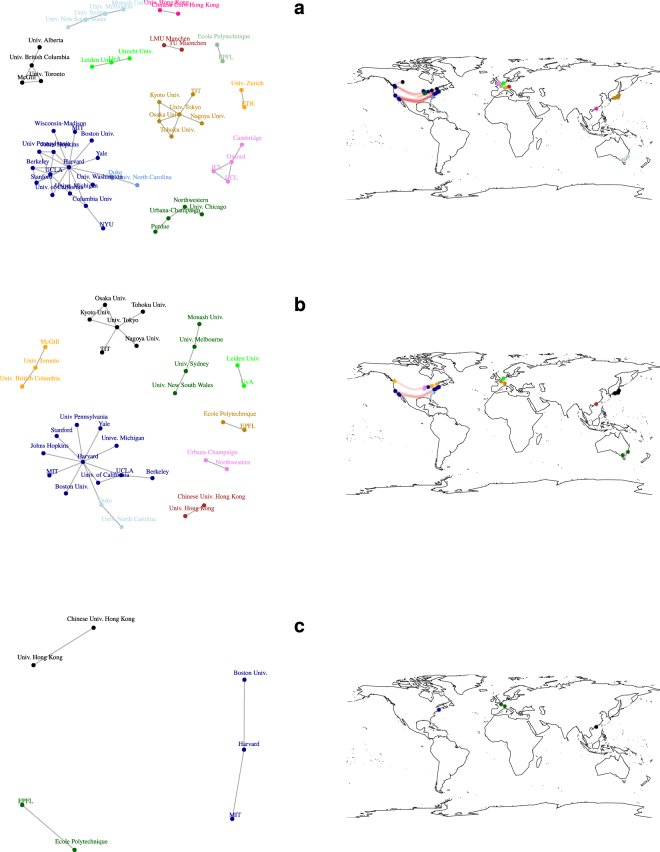
Snapshots of network topology for different thresholds: (**a**) *w*_*T*_ = 400, (**b**) *w*_*T*_ = 500 and (**c**) *w*_*T*_ = 1000. The colors of vertices correspond to the assignment from a community detection algorithm (fast greedy modularity optimization algorithm^47^) and therefore they can change from one panel to another. Plots were created combining open-source packages ${\mathtt{i}}{\mathtt{g}}{\mathtt{r}}{\mathtt{a}}{\mathtt{p}}{\mathtt{h}}$^31^ (nodes and links) and ${\mathtt{m}}{\mathtt{a}}{\mathtt{p}}{\mathtt{s}}$^48^ (world map) for ${\mathtt{R}}$ language^30^.

The above described non-trivial behavior of quantities *r*, *C* and 〈*l*〉 and *S* cannot be the sole cause of the relations presented in Fig. 3b although a high number of points with *w*_*T*_ ≈ 100 can be responsible for some of these effects. It seems that there has to be another phenomenon leading to such an effect. Using ${\mathtt{R}}$’s^30^ package ${\mathtt{igraph}}$^31^ we visualize connections between universities and community structure (denoted by color) for different values of *w*_*T*_. The results for *w*_*T*_ = 100, 200, 300 and *w*_*T*_ = 400, 500, 1000 are shown in Figs 6 and 7, providing an input for further analysis. For *w*_*T*_ = 100 (Fig. 6a) the network is still percolated, i.e., it is possible to reach any node from another one; over that value a separation occurs - Chinese, Australian and Singapore, Japanese, Danish and Swedish as well as Swiss universities all form separate clusters. This observation is connected with large loss of *S* in Fig. 5f. The remaining giant cluster is built out of American, Canadian, British, Dutch, and German universities (Fig. 6b). This is the area where both average path length 〈*l*〉 and assorativity *r* take their maximal values. For *w*_*T*_ = 300 we witness the separation between US and British universities and from now on (with small exceptions) different clusters can be described as connected to different countries (or even smaller administrative units as English and Scottish universities are separated). Further plots depict progressing decay of connections between the universities that form either star-like structures (Japanese, Canadian, English and American in Fig. 7a,b) or ultimately chains (Fig. 7c).

**Figure 8:**
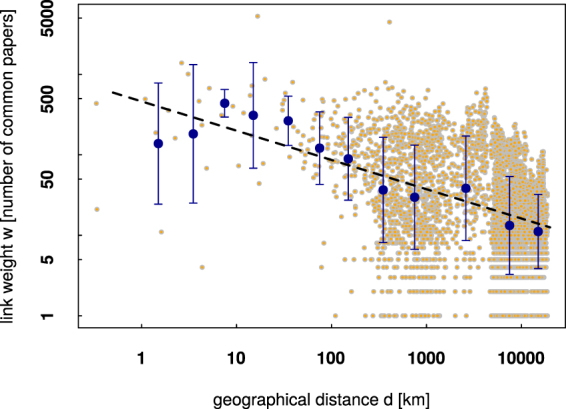
Link weight *w* vs the geographical distance *d* between universities in a double logarithmic scale. Orange-gray circles are raw data while the blue circles with error bars come from logarithmic binning of data with. Dashed line is a power-law fit *w* = *Ad*^*α*^ with *A* = 461.0 ± 1.4 and *α* = −0.364 ± 0.058.

A possible explanation to this phenomenon is in the geographical distance between the universities. In fact, Fig. 8 supports partially this assumption. The number of publications between universities *i* and *j* can be fitted with a decreasing power-law function of the geographical distance between them. The gap around *d* = 5000 is most probably caused by the presence of continents. Similar results regarding the role of geographical distance in science were obtained in previous studies^25,32^. On the other hand the error bars in Fig. 8 give evidence that for relatively short distances (*d* ∈ [1; 300] km) the number common papers can be considered constant. This in turn would support the hypothesis of country-driven rather than geographically-driven collaboration. A lower than expected value of collaboration for shorter distances could also have its origin in the fact that usually there is lack of universities of the same scientific profile in the direct vicinity.

## Conclusions

Our results indicate that even such fundamental and straightforward analysis as calculation of correlation coefficient between position of the university in the ranking and the number of papers published by its employees may reveal some non-trivial relationships. Although it would be natural to expect strictly negative correlation (i.e., the more you publish the higher rank you acquire) our analysis shows several scientific disciplines such as *Agricultural Engineering*, *Horticulture* or *Hospitality*, *Leisure*, *Sport & Tourism* where this is not the case. For the whole set of examined scientific categories we found a log-linear relationship between correlation and the number of papers. Intriguingly this relation breaks down when the most reliable correlations (i.e., most significant statistically) are selected. This study also underlines the differences among specific science areas — our PCA results give a clear picture that the separation between natural, medical and social sciences really takes place.

The second part of the paper is devoted to network analysis of the collaboration among 100 best universities. We used the concept of weight threshold to obtain several slices of the original weighted network at different levels of collaboration intensity. Treating the threshold as a control parameter we were able to track such network observables as assortativity revealing its rich behavior. Our analysis shows that the scientific collaboration is highly embedded in the physical space - it seems that the key aspect that governs the number of common publications is the geographical vicinity of the universities which confirms previous observations^25,32^. On the other hand the dependence of network properties on the weight threshold cannot be explained just by using geographical distance rationale suggesting rather country-driven collaboration.

## Discussion

The problem of the role of scientific categories and relations among them has intrigued the greatest minds of the past century. Lately, Dias *et al*.^33^ have explicitly quoted Karl Popper’s *The Nature of philosophical problems and their roots in science*^34^ where this great philosopher had questioned the traditional identification of scientific disciplines, convinced instead that one should rather look at cognitive and social aspect thereof. Dias *et al*. follow this trail by comparing coincidences among disciplines retrieved by (i) classification given by experts^29^, (ii) Jaccard-like coefficient for citations and (iii) language-based Jensen-Shannon measure of dissimilarity^35,36^ in articles’ abstracts. The same aspect, although in much more indirect way, has been lately addressed by one of us, arguing that scientific segregation is visible even while examining relations between text length (or emotional content) and citation patterns^17^. While these considerations may seem to be academic (e.g., detecting similarities among disciplines that are “obviously” similar) they earn an additional dimension when treated as a dynamical process. Given the masses of data the usage *unsupervised* methods that require no manual classification of documents is the best choice to track the evolution of science. In this way such phenomena as convergence and divergence of specific disciplines^33^, life cycles of paradigms^37^ or inheritance of scientific memes^20^ can be instantly spotted. When used for temporal data, our analysis of principal components basing on the number of published papers could also serve as an index for changing relations among disciplines. In particular, one may use it as indicator of the interest a certain scientific area gains over the years. It is possible to spot the emergence of certain trends in science and, in effect, react by for example establishing a new direction of research in the university.

Geographical distances among the nodes of the network usually come in the form of Tinbergen’s gravity model^38^. Manifestations of spatial embedding of networks^39^ are truly omnipotent, ranging from the original inter-country trade^40,41^ through inter-city telecommunication flows^42^ and online friendship^43^ to active protesters^44^. In the case of scientific collaboration Pan *et al*. show a clear preference for researchers to seek partners in their geographical proximity^25^, however underlining that the very form of the gravity model (i.e., a power law) does not forbid long-distance interactions. In this study we restricted ourselves to only top universities showing which particular links break up first. Although the geographical proximity is an important factor, the results clearly show that in the case of small distances the connections are not formed distance-wise but rather country-wise. Moreover it also seems that the choice of data handling method (absolute values vs. normalized one) can play a crucial role: the description as well as Figs S2 and S3 in the Supplementary Material reveal a strong clustering between continents for the normalized data.

## Methods

### Dataset

We used two prominent data providers: QS World University Ranking^45^ and Web of Science^46^ service. The first dataset consisted of 100 best universities ranked in the year 2009. The second dataset was obtained by querying the database of years 2008**–**2009 for publications coming from one of the above mentioned universities and store information about so-called subject category (i.e., the scientific category) and affiliation of co-authors. The obtained matrices **P**_*ij*_ (100 universities by 181 categories) and **C**_*ij*_ (100 by 100 universities) that were created on-the-fly without physically saving partial data contain, respectively, 1363821 and 496684 papers.

### Abbreviations

The seemingly straightforward procedure of querying for a specific university name encounters some problems that could have a strong impact on the further results. Web of Science has a set of abbreviations commonly used for searching such as *Univ* for “University” or *Coll* for “College”. Moreover it is essential to notice that one has to form a very specific query in order to get rid of severe mistakes. Table 3 shows an exemplary list of the search universities together with the exact search phrase that had to be used.

**Table 3:** University names and search queries.

Rank	University	Search query
1	Harvard University	Harvard Univ
2	University of Cambridge	Univ Cambridge
4	UCL University College London	UCL
10	California Institute of Technology	Caltech
73	Washington University in St. Louis	Washington Univ + St Louis
98	Ludwig-Maximilians-Universität München	Univ Munich | Tech Univ Munich

### Ambiguity of queries

The ‘Search’ field is a search key that we use to associate with the authors of the publications and it can consist of one of the operators: + stands for *AND* operator in Boolean logic and | stands for *NOT* operator in Boolean logic. These operators are used to clearly assess the origin of the publication. Table 2 shows that using just the names of universities from the list (first column) would lead in the case of number 98 to obtaining publications of both *Technical University in Munich* and *University of Munich*, instead of just the latter. To avoid this problem one has to insert a query *Univ Munich* | *Tech Univ Munich* that ensures achieving proper results. On the other hand for instance for the case shown as number 78, it was not sufficient to enter *Washington Univ*, as there are many universities with such an abbreviation; it was necessary to add *St*. *Louis* in the query text.

### Network analysis

Clustering coefficient *C*_*i*_ for node *i* is defined as the number of existing links among its nearest neighbors *e*_*i*_ (i.e., nodes to which it has links) divided by the total number of possible links among them *k*_*i*_(*k*_*i*_ − 1)/2

1 $${C}_{i}=\frac{2{e}_{i}}{{k}_{i}({k}_{i}-1)}$$

The total clustering coefficient for the whole network is calculated as the average over all *C*_*i*_.

Assortativity coefficient *r* defined by

2 $$r=\frac{\frac{1}{E}\,{\sum }_{i}\,{j}_{i}{k}_{i}-{[\frac{1}{2E}{\sum }_{i}({j}_{i}+{k}_{i})]}^{2}}{\frac{1}{2E}({\sum }_{i}\,{j}_{i}^{2}+{k}_{i}^{2})-{[\frac{1}{2E}{\sum }_{i}({j}_{i}+{k}_{i})]}^{2}}$$

where i goes over all edges in the network. The coefficient is in the range [−1; 1], *r* = 1 means that the highly connected nodes have the affinity to connect to other nodes with high *k*_*i*_ while *r* = −1 happens when highly connected nodes tend to link to nodes with very low *k*_*i*_.

Average shortest path 〈*l*〉 is calculated as the average value of shortest distance (measured in the number of steps) between all pairs of nodes *i*, *j* in the network.

## Electronic supplementary material

Supplementary Information

## References

[1] Kuhn, T. S. *The Structure of Scientific Revolutions* (University of Chicago Press, 1996).

[2] Popper, K. *The Logic of Scientific Discovery* (Routledge, 2002).

[3] Lakatos, I. *The Methodology of Scientific Research Programmes* (Cambridge University Press, 1980).

[4] Feyerabend, P. *Against method* (Verso, 2010).

[5] Merton RK (1968). The matthew effect in science. Science.

[6] King DA (2004). The scientific impact of nations. Nature.

[7] Hirsh JE (2005). An index to quantify an individual’s scientific research output. Proceedings of the National Academy of Sciences of the United States of America.

[8] Radicchi F, Fortunato S, Castellano C (2008). Universality of citation distributions: Toward an objective measure of scientific impact. Proceedings of the National Academy of Sciences of the United States of America.

[9] Radicchi F, Fortunato S, Markines B, Vespignani A (2009). Diffusion of scientific credits and the ranking of scientists. Physical Review E.

[10] Petersen AM, Wang F, Stanley HE (2010). Methods for measuring the citations and productivity of scientists across time and discipline. Physical Review E.

[11] Radicchi F, Castellano C (2011). Rescaling citations of publications in physics. Physical Review E.

[12] Mzaloumian A, Young-Ho E, Helbing D, Lozano S, Fortunato S (2011). How citation boosts promote scientific paradigm shift and nobel prizes. PLoS One.

[13] Fronczak P, Fronczak A, Hołyst JA (2007). Analysis of scientific productivity using maximum entropy principle and fluctuation-dissipation theorem. Physical Review E.

[14] Sinatra R, Wang D, Deville P, Song C, Barabási A-L (2016). Quantifying the evolution of individual scientific impact. Science.

[15] Moed HF (2017). A critical comparative analysis of five world university rankings. Scientometrics.

[16] Bollen J, Van de Sompel H, Hagberg A, Chute R (2009). A principal component analysis of 39 scientific impact measures. PLoS One.

[17] Sienkiewicz J, Altmann EG (2016). Impact of lexical and sentiment factors on the popularity of scientific papers. Royal Society Open Science.

[18] Patience GS, Patience CA, Blais B, Bertrand F (2017). Citation analysis of scientific categories. Heliyon.

[19] Perc M (2013). Self-organization of progress across the century of physics. Scientific Reports.

[20] Kuhn T, Perc M, Helbing D (2014). Inheritance patterns in citation networks reveal scientific memes. Physical Review X.

[21] Narin F, Stevens K, Whitlow ES (1991). Scientific co-operation in europe and the citation of multinationally authored papers. Scientometrics.

[22] Glänzel W, Schubert A, Czerwon HJ (1999). A bibliometric analysis of international scientific cooperation of the european union (1985**–**1995). Scientometrics.

[23] Jones BF, Wuchty S, Uzzi B (2008). Multi-university research teams: Shifting impact, geography, and stratification in science. Science.

[24] Börner K, Penumarthy S, Meiss M, Ke W (2006). Mapping the diffusion of scholarly knowledge among major US research institutions. Scientometrics.

[25] Pan RK, Kaski K, Fortunato S (2012). World citation and collaboration networks: uncovering the role of geography in science. Scientific Reports.

[26] Chen RH-G, Chen C-M (2016). Visualizing the world’s scientific publications. Journal of the Association for Information Science and Technology.

[27] Barabási AL, Albert R (2002). Statistical mechanics of complex networks. Reviews of Modern Physics.

[28] Chmiel A, Sienkiewicz J, Suchecki K, Hołyst JA (2007). Networks of companies and branches in poland. Physica A.

[29] Oecd classification. https://www.oecd.org/science/inno/38235147.pdf (Accessed on 7th July 2017).

[30] R Core Team. *R*: *A Language and Environment for Statistical Computing*. R Foundation for Statistical Computing, Vienna, Austria, https://www.R-project.org/ (2017).

[31] Csardi, G. & Nepusz, T. The igraph software package for complex network research. *InterJournal* Complex Systems, 1695, http://igraph.org (2006).

[32] Hennemann, S., Rybski, D. & Liefner, I. The myth of global science collaboration patterns in epistemic communities. *Journal of Informetrics* **6**, 217–225 (2012).

[33] Dias L, Gerlach M, Scharloth J, Altmann EG (2018). Using text analysis to quantify the similarity and evolution of scientific disciplines. Royal Society Open Science.

[34] Popper KR (1952). The nature of philosophical problems and their roots in science. The British Journal for the Philosophy of Science.

[35] Gerlach M, Font-Clos F, Altmann EG (2016). Similarity of symbol frequency distributions with heavy tails. Physical Review X.

[36] Altmann EG, Dias L, Gerlach M (2017). Generalized entropies and the similarity of texts. Journal of Statistical Mechanics: Theory and Experiment.

[37] Chavalarias D, Cointet J-P (2013). Phylomemetic patterns in science evolution—the rise and fall of scientific fields. PLoS One.

[38] Squartini, T. & Garlaschelli, D. *Jan Tinbergen*’*s Legacy for Economic Networks*: *From the Gravity Model to Quantum Statistics*, 161–186 (Springer International Publishing, Cham 2014).

[39] Barthélemy M (2011). Spatial networks. Physics Reports.

[40] Kaluza P, Kölzsch A, Gastner MT, Blasius B (2010). The complex network of global cargo ship movements. Journal of The Royal Society Interface.

[41] Karpiarz M, Fronczak P, Fronczak A (2014). International trade network: Fractal properties and globalization puzzle. Physical Review Letters.

[42] Krings G, Calabrese F, Ratti C, Blondel VD (2009). Urban gravity: a model for inter-city telecommunication flows. Journal of Statistical Mechanics: Theory and Experiment.

[43] Liben-Nowell D, Novak J, Kumar R, Raghavan P, Tomkins A (2005). Geographic routing in social networks. Proceedings of the National Academy of Sciences of the United States of America.

[44] Traag V, Quax R, Sloot P (2017). Modelling the distance impedance of protest attendance. Physica A.

[45] Top Universities. https://www.topuniversities.com/ (Accessed on 7th July 2017).

[46] Web of Science. http://clarivate.com/scientific-and-academic-research/research-discovery/web-of-science/ (Accessed on 7th July 2017).

[47] Clauset A, Newman MEJ, Moore C (2004). Finding community structure in very large networks. Physical Review E.

[48] code by Richard, A. & Becker, O. S. version by Ray Brownrigg. Enhancements by Thomas P. Minka, A. R. W. R. & Deckmyn, A. *maps*: *Draw Geographical Maps*, https://CRAN.R-project.org/package=maps, R package version 3.2.0 (2017).

