# Supplementary material for: Categorical and Geographical Separation in Science
Source: Sci Rep. 2018 May 29;8:8253. doi: 10.1038/s41598-018-26511-4 (PMC5974177; doi:10.1038/s41598-018-26511-4)
Supplement: Supplementary file 1 — Supplementary Information [file 41598_2018_26511_MOESM1_ESM.pdf]

Categorical and Geographical Separation in Science - Supplementary Information

Julian Sienkiewicz[1], **Krzysztof Soja**[1], **Janusz A. Hołyst**[1,2], **and Peter M. A. Sloot**[2,3,4*]

[1]Faculty of Physics, Center of Excellence for Complex Systems Research, Warsaw University of Technology, Warsaw, Poland
[2]National Research University of Information Technologies, Mechanics and Optics (ITMO), Saint Petersburg, Russia
[3]Institute for Advanced Study, University of Amsterdam, Amsterdam, The Netherlands
[4]Nanyang Technological University, Singapore, Singapore
[*]p.m.a.sloot@uva.nl

ABSTRACT

In this document we present some additional analysis regarding rank prediction by ordinal regression model and null model for networking thresholding procedure. Moreover we show a simple variation of main results shown in the paper: instead of taking the absolute values of the number of papers published in different categories and between different universities, we use normalized values.

Ordinal regression analysis of rank

An alternative way to examine the correlations between the rank and number of papers is to assume that QS rank emerges as a consequence of the number of papers published by each university in different scientific categories. In this way one can treat the rank as an ordinal variable and perform ordinal regression analysis (see, e.g.,[1]). We split our original dataset in order to have a training set (year 2008) and test set (year 2009) and perform ordinal regression using `MASS`[2] package for `R` language[3]. The results are shown in Fig. 1, proving that in this case the methods leads to an overfitting for year 2008 (when this dataset is used both as a training and test set) and brings rather unreliable outcomes when used to predict rank in year 2009.

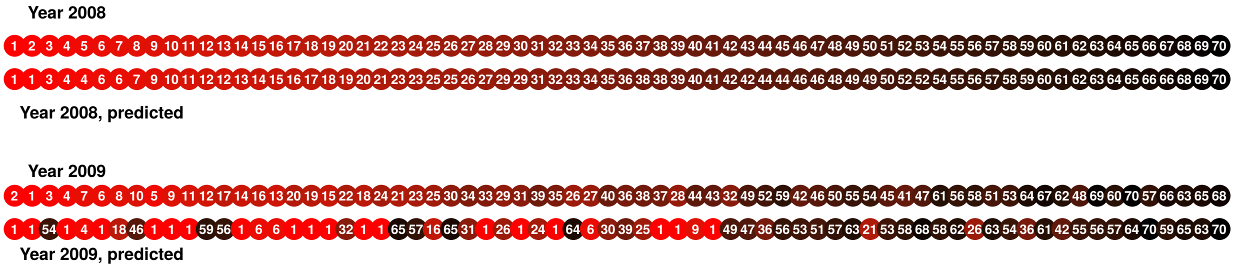

Figure 1. Ordinal regression analysis. Two top plots show rank in year 2008 and ordinal regression prediction. Bottom plots give the same in case of year 2009. The rank is shown as numbers 1–70 and also color-coded: red is the highest and black — the lowest.

Network observables as a function of weight threshold

Figure 5 in the main text presents several network statistics as a function of the weight threshold w_T. A possible naive null model could be formulated in the following way: resample the weights of the network and perform the thresholding procedure again. After several realizations of such an algorithm (here we use $M = 100$) one would obtain a statistical reference plotting mean value of the considered observables against w_T. Such results are presented in Fig. 2 underlining differences between the actually observed values and the ones obtained from weight reshuffling. The most prominent example is the assortativity coefficient r.

Rank–number correlations for categories

As an alternative approach to the one presented in the main text we use relative numbers for $\mathbf{P}_{ij}$ defined as

$$\hat{\mathbf{P}}_{ij} = \frac{\mathbf{P}_{ij}}{\sum_i \mathbf{P}_{ij}}. \tag{1}$$

In this way each row of $\hat{\mathbf{P}}_{ij}$ presents the *relative* number of papers published by university i in a given scientific category j. Owing to this fact high numbers papers of leading universities do not diminish larger impact exerted by smaller unities (e.g., university A has 1000 papers in category X but the total number of its articles is 100000 while university B with a total number of 100 papers publishes all of them in category A). As a result we observe (Fig. 3) the absence of "powerful" categories such as *Physics* and appearance of such ones as *Geography* or *Ergonomics* missing in the main paper. There is considerable amount of categories with statistically significant positive correlations. Intriguingly the category of *Multidisciplinary Sciences* is still the most negatively correlated data. A possible interpretation of this fact is shown in the top row of Fig. 4: in the case of such underrepresented (in the sense of numbers of papers) scientific categories as *Food Science and Technology* low-ranked universities, characterized in general by lower total number of papers are more prone to higher fluctuations of the number of articles. This in turn can lead to an appearance of higher values in the relative number of papers for such universities. On the other hand in case of high-ranked bodies such fluctuations are negligible due to high total numbers of papers. In effect correlation coefficients can be significantly positive. Such a situation is not observed for such categories as *Multidisciplinary Sciences* (bottom row).

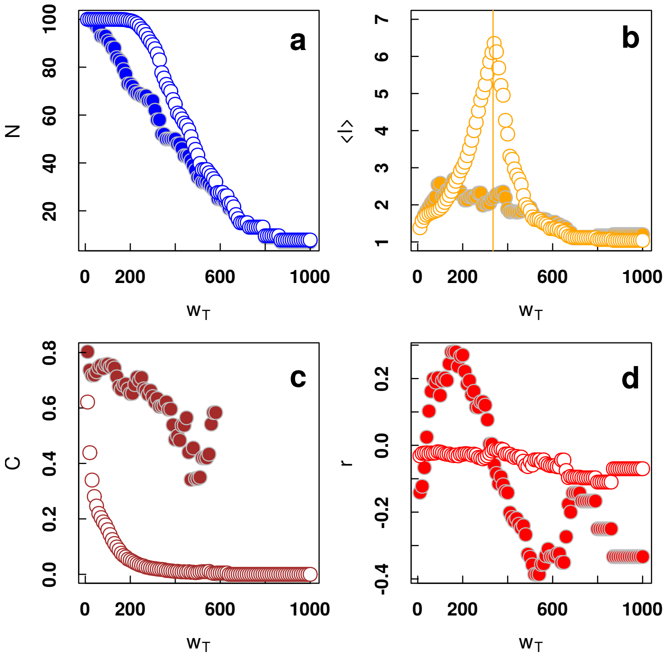

Figure 2. Network observables. Comparison of simulated collaboration networks observables (empty symbols) and the actual observed values (full symbols, Fig. 5 in the main text) as functions of weight threshold w_T : (a) number of nodes N, (b) average shortest path $\langle l \rangle$; the vertical line marks the point for which $\langle k \rangle = 1$, (c) clustering coefficient C, (d) assortativity coefficient r.

Network visualization

The collaboration matrix $\mathbf{C}_{ij}$ is transformed into

$$\hat{\mathbf{C}}_{ij} = \frac{\mathbf{C}_{ij} \sum_{ij} \mathbf{C}_{ij}}{\sum_i \mathbf{C}_{ij} \sum_j \mathbf{C}_{ij}}. \tag{2}$$

Unlike $\mathbf{C}_{ij}$ in the main manuscript this ensures that $\hat{\mathbf{C}}_{ij}$ is a relative measure and has the form of Pointwise Mutual Information (without the logarithm). Thus $\hat{\mathbf{C}}_{ij} = 1$ indicates independence between common papers of i and j. In this way all values $\hat{\mathbf{C}}_{ij} > 1$ point to intensive collaboration between i and j. Following the thresholding procedure described in the main part of the paper we create several snapshots of the collaboration network for $w_T = 2, 3, 5$ (Fig. 5) and $w_T = 10, 20, 50$ (Fig. 6). In this setting we observe strikingly different situation that the one shown in the main paper — the percolation cluster survives very long even when a lot links are cut off (see Fig. 5c). Although clusters connected to single countries emerge the links between different continents are still sustained and the bridge connecting US and Europe is visible even for $w_T = 20$.

References

1. Winship, C. & Mare, R. D. Regression models with ordinal variables. *American Sociological Review* **49**, 512–525 (1984).

2. Venables, W. N. & Ripley, B. D. *Modern Applied Statistics with S* (Springer, New York, 2002), fourth edn. URL http://www.stats.ox.ac.uk/pub/MASS4. ISBN 0-387-95457-0.

3. R Core Team. *R: A Language and Environment for Statistical Computing*. R Foundation for Statistical Computing, Vienna, Austria (2017). URL https://www.R-project.org/.

4. Clauset, A., Newman, M. E. J. & Moore, C. Finding community structure in very large networks. *Phys. Rev. E* **70**, 066111 (2004). URL https://link.aps.org/doi/10.1103/PhysRevE.70.066111.

5. Csardi, G. & Nepusz, T. The igraph software package for complex network research. *InterJournal* **Complex Systems**, 1695 (2006). URL http://igraph.org.

6. code by Richard A. Becker, O. S., version by Ray Brownrigg. Enhancements by Thomas P Minka, A. R. W. R. & Deckmyn., A. *maps: Draw Geographical Maps* (2017). URL https://CRAN.R-project.org/package=maps. R package version 3.2.0.

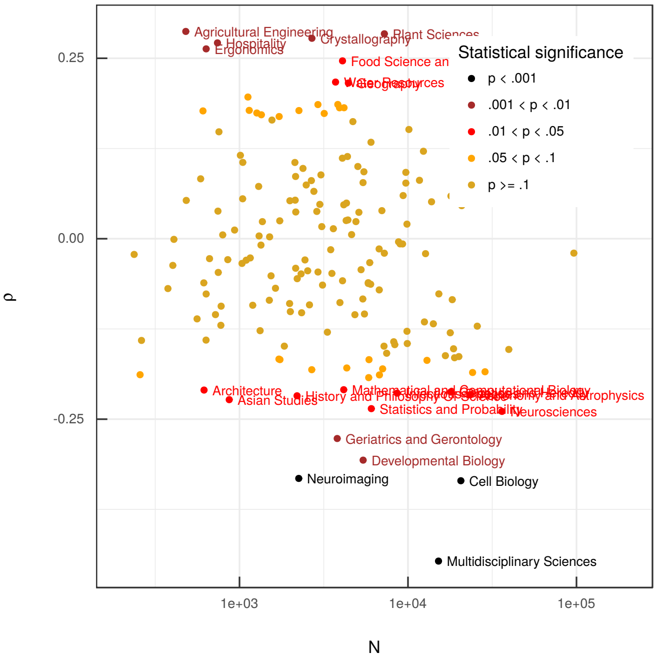

Figure 3. Correlations coefficients. Each data point represents separate scientific category and gives Spearman's correlation coefficient between the rank of the university and the ranked number of papers in this category (shown as X-axis). The colors of points reflect statistical significance of the measure (see legend) and category names are shown only for the most significant points (p-value $<$ .05).

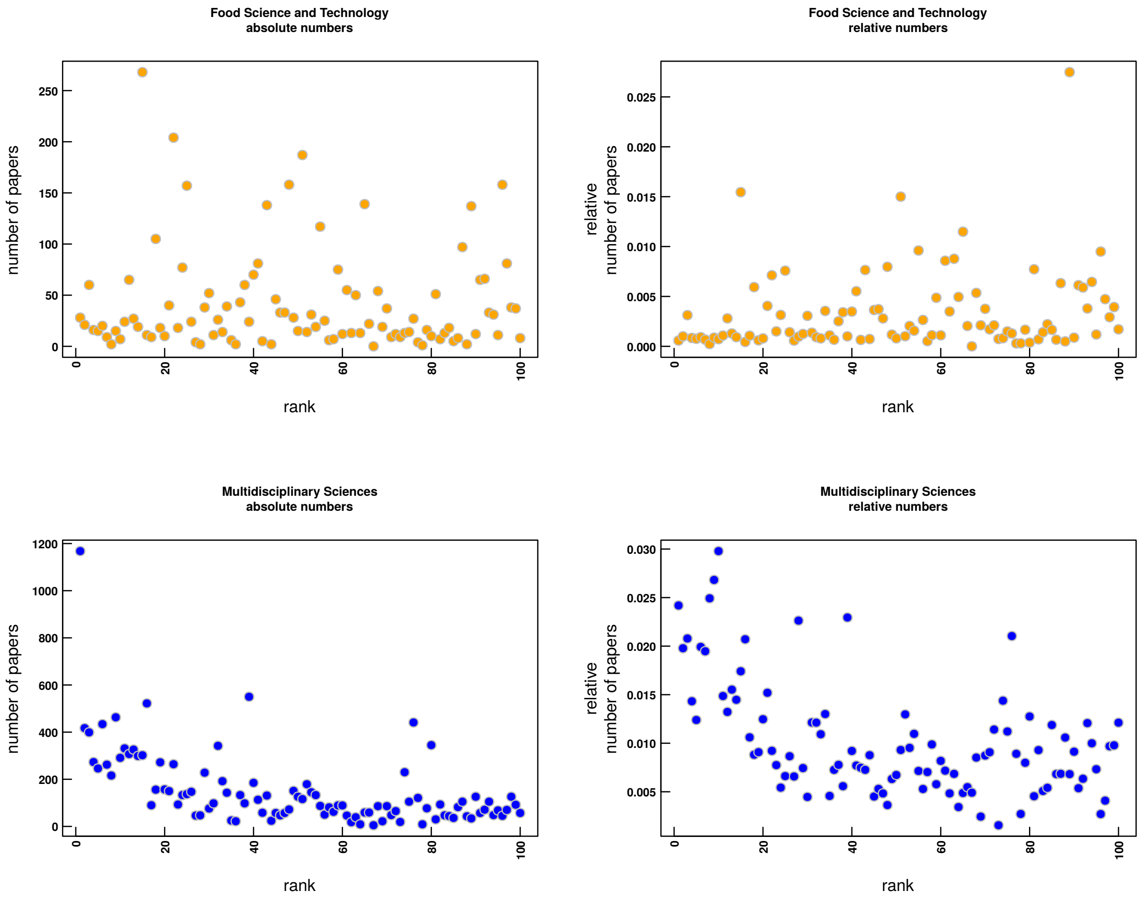

Figure 4. Comparison between absolute and relative number of papers. Top row: absolute (left) and relative (right) number of papers for *Food Science and Technology* vs. rank. Bottom row: absolute (left) and relative (right) number of papers for *Multidisciplinary Sciences* vs. rank.

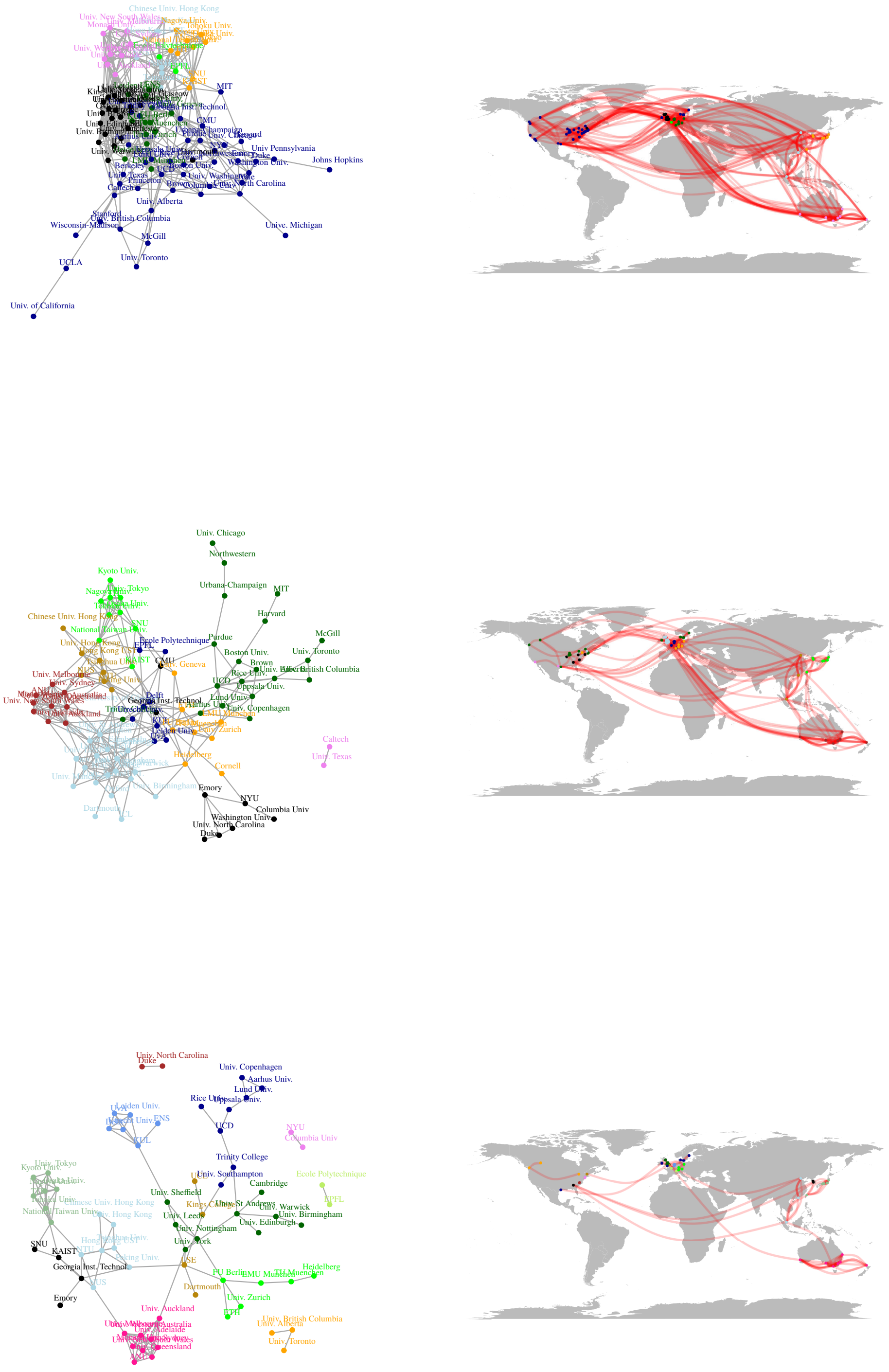

Figure 5. Snapshots of network topology for different thresholds: (a) $w_T = 2$, (b) $w_T = 3$ and (c) $w_T = 4$. The colors of vertices correspond to the assignment from a community detection algorithm (fast greedy modularity optimization algorithm[4]) and therefore they can change from one panel to another. Plots were created combining open-source packages `igraph`[5] (nodes and links) and `maps`[6] (world map) for `R` language[3].

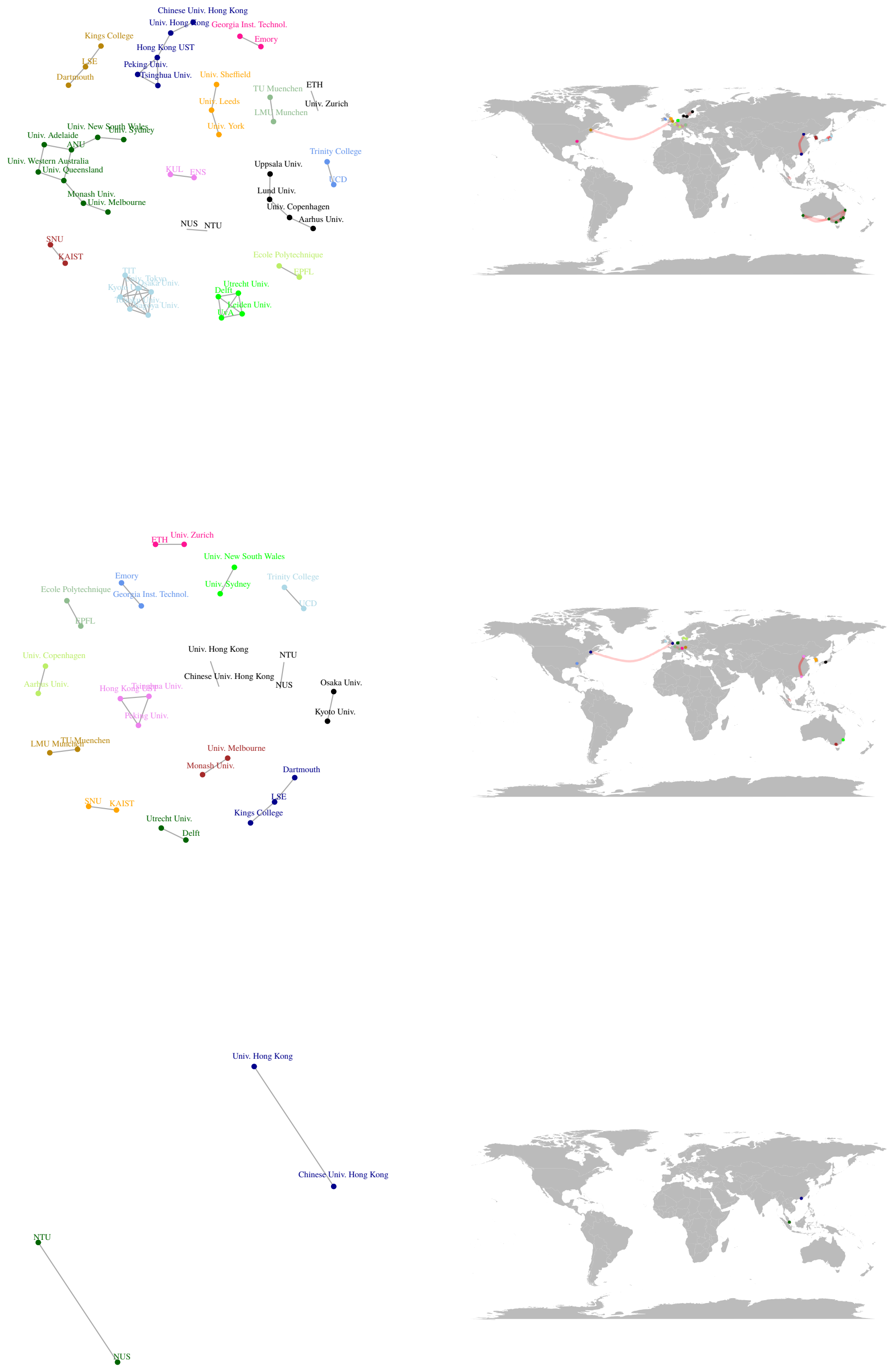

Figure 6. Snapshots of network topology for different thresholds: (a) $w_T = 10$, (b) $w_T = 20$ and (c) $w_T = 50$. The colors of vertices correspond to the assignment from a community detection algorithm (fast greedy modularity optimization algorithm[4]) and therefore they can change from one panel to another. Plots were created combining open-source packages `igraph`[5] (nodes and links) and `maps`[6] (world map) for `R` language[3].
